# *Skrjabingylus chitwoodorum* in a rabies-positive striped skunk in Texas

**DOI:** 10.1177/10406387241293421

**Published:** 2024-11-07

**Authors:** Sarah Myers, Brianne Taylor, Ragan Wilson, Shannon Caseltine, Ruth C. Scimeca

**Affiliations:** Department of Veterinary Pathobiology, Oklahoma State University, Stillwater, OK, USA; Department of Veterinary Pathobiology, Oklahoma State University, Stillwater, OK, USA; Department of Veterinary Pathobiology, Oklahoma State University, Stillwater, OK, USA; Oklahoma Animal Disease Diagnostic Laboratory, Oklahoma State University, Stillwater, OK, USA; Department of Veterinary Pathobiology, Oklahoma State University, Stillwater, OK, USA

**Keywords:** *Mephitis mephitis*, rabies, *Skrjabingylus chitwoodorum*, skunk cranial worm

## Abstract

We describe here a case of the sinus roundworm, *Skrjabingylus chitwoodorum*, found incidentally in a rabies-positive striped skunk (*Mephitis mephitis*) in Texas, USA. Skunks serve as a natural definitive host for this metastrongylid nematode in North America, in which infections result in observable damage to the host cranium, where adult parasites reside. Additionally, skunks are considered the primary reservoir of rabies in Texas. In November 2022, the animal was discovered in northern Texas displaying neurologic signs before euthanasia and submission to the Oklahoma Animal Disease Diagnostic Laboratory for rabies testing. Direct fluorescent antibody testing indicated that the animal was rabies-positive, and, upon tissue collection, numerous adult nematodes were recovered from the cranium and identified as *S. chitwoodorum* by morphology and amplification of the mitochondrial cytochrome c oxidase subunit I gene. Histologically, we found lymphohistiocytic meningitis in several loci and chronic sinusitis rostral to the cribriform plate. Due to behavioral abnormalities, we additionally tested for *Toxoplasma gondii* via PCR, but no parasite DNA was detected. Concurrent infection by *S. chitwoodorum* and rabies virus may contribute to neurologic signs in skunks.

*Skrjabingylus chitwoodorum* is a metastrongylid nematode that primarily parasitizes the frontal sinuses of the striped skunk (*Mephitis mephitis*), leading to neurologic signs and profound skull deformities.^12,14^ Due to the predilection for specific host species and anatomic location, this nematode is commonly referred to as the skunk cranial worm or sinus roundworm.^7,12^ Six species are described within the genus *Skrjabingylus*, including *S. chitwoodorum*, *S. lutrae*, *S. nasicola*, *S. petrowi*, *S. ryjikovi*, and *S. santaceciliae*; however, only *S. chitwoodorum* and *S. santaceciliae* have been described from *M. mephitis* in North America.^2,16^ Although all occur in the frontal sinuses of mustelids and mephitids, *S. chitwoodorum* can be differentiated from other species in the genera given its larger body size and morphology of male copulatory spicules, which are up to 4 times longer than those of other species.^16^

Though primarily a parasite of the striped skunk, *S. chitwoodorum* has also been reported in spotted skunks (*Spilogale* spp.), with descriptions of more severe lesions in the cranium compared to striped skunks.^11,14^ Originally described in Oklahoma in 1939, *S. chitwoodorum* has since been reported throughout the United States, Canada, and Mexico where skunks are present.^13^ The life cycle of *S. chitwoodorum* begins when first-stage larvae are passed in the feces of the definitive host and enter a terrestrial gastropod intermediate host. The first-stage larvae develop into infective third-stage larvae within terrestrial snail intermediate hosts before being consumed by a paratenic host or the definitive host. Paratenic hosts include frogs, snakes, and rodents (Fig. 1). Within the skunk definitive host, the third-stage larvae molt twice into fifth-stage larvae in the gut before migrating through the intestinal wall and following peripheral nerves to the CNS.^17^ Larvae will continue migration in the spinal subarachnoid space to the front of the brain until penetrating the cribriform plate and reaching the frontal sinuses, where the adults will mature.^1^ Mature adults release first-stage larvae formed in utero into the sinuses where they reach the back of the pharynx and are swallowed. Maturation of these worms in the frontal sinus can occur as early as 6 d after ingestion and leads to incoordination, lethargy, and progressive cranial osteologic deformity in the skunk definitive host.^14^ Severe cranial lesions and increased numbers of adult worms have been associated with older skunks; in addition, perforation of the frontal sinuses can occur with advanced infections.^13,14^ Skunks infected with *S. chitwoodorum* can sometimes display behavioral abnormalities.^18^

**Figure 1.**
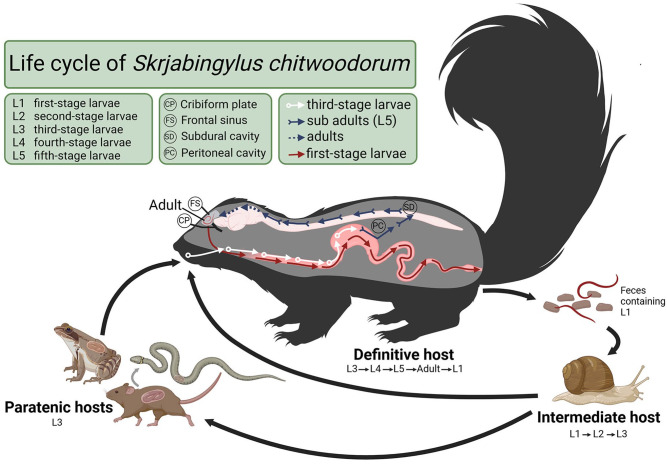
Life cycle of *Skrjabingylus chitwoodorum* in skunks, created in BioRender.

In November 2022, a skunk was roaming disoriented in the town of Darrouzett, TX, USA during the daytime. Because of the odd behavior, the skunk was euthanized, and the cranium was submitted for rabies testing to the Oklahoma Animal Diseases Diagnostic Laboratory (OADDL; Oklahoma State University, Stillwater, OK, USA). In addition to rabies virus (RABV; *Rhabdoviridae*, *Lyssavirus rabies*), the protozoan parasite *Toxoplasma gondii* has been reported to cause behavioral abnormalities in different hosts.^10^ Thus, we tested for *T. gondii* as well as RABV.

The skunk cranium had extensive meningoencephalitis and contained several nematodes (Fig. 2A). The specimens recovered were bright red, with a thick transparent cuticle containing annular ridges (Suppl. Fig. 1) and a long and muscular esophagus (Suppl. Fig. 2). The female worms were ~4 cm long; males were smaller, with an average length of 2.6 cm. Females had a short tail with a subterminal protuberance (Fig. 2B). Males were darker than females, with a posterior end that had 6 rays and spicules similar in size and shape (650 µm average; Fig. 2C), each terminating posteriorly in a leaf-like, dorsoventrally flat, expansion.

**Figure 2.**
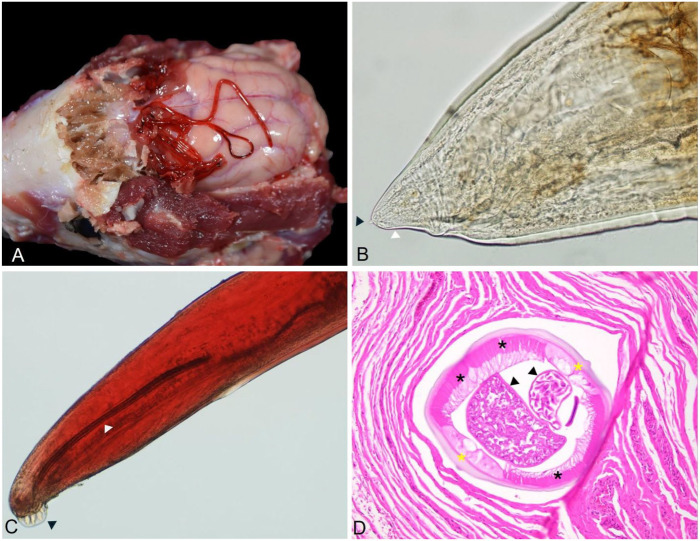
Adult *Skrjabingylus chitwoodorum* nematodes from a skunk. **A.** Cranium with meningoencephalitis and nematodes. **B.** Caudal end of *S. chitwoodorum* female with short tail (white arrowhead) and a subterminal protuberance (black arrowhead). **C.** Caudal end of *S. chitwoodorum* male with 6 rays (black arrowhead) and long spicules (white arrowhead). **D.** Transverse section of *S. chitwoodorum* within the skeletal musculature over the frontal sinus; ~1,000-µm diameter with 10–25-µm eosinophilic, smooth-to-undulating cuticle, hypodermis with lateral chords (yellow asterisks), celomyarian-polymyarian musculature (black asterisks), and pseudocelom containing reproductive tracts (black arrowheads).

We isolated DNA from parasite specimens and host brain tissue (Quick DNA miniprep plus kit; Zymo), following the manufacturer’s instructions. We used the parasite DNA to confirm our morphologic identification of the nematodes by a PCR assay, and the DNA from brain tissue to test for *T. gondii* DNA.

We tested parasite DNA by PCR, using the primers NemF2_t1 (ARAGATCTAATCATAAAGATATYGG) and NemR2_t1 (AWACYTCWGGRTGMCCAAAAAAYCA),^3,20^ which amplify a region of the cytochrome c oxidase subunit I (*COI*) gene from vertebrate parasitic nematodes. We used 25 µL of DNA polymerase (GoTaq; Promega), 1 µL of each primer, 2 µL of DNA sample, and 21 µL of DNase-free water for the PCR reaction. The PCR conditions were set as follows: pre-denaturation at 94°C for 3 min followed by 35 cycles of denaturation at 94°C for 30 s, an annealing temperature of 51°C for 45 s, and extension at 72°C for 1 min, followed by a final extension at 72°C for 7 min. The PCR products were separated in a 1.5% agarose gel and observed with nucleic acid stain (GelRed; Biotium).

We purified the amplicons with a genomic DNA purification kit (GeneJET; Thermo Fisher), according to the manufacturer’s instructions, followed by submission to Eurofins Genomics (Louisville, KY) for Sanger sequencing. We compared the sequences in BLAST (https://blast.ncbi.nlm.nih.gov/Blast.cgi) and aligned them using Geneious Prime software v.2023.0.4 (Dotmatics).

For histology purposes, we collected selected samples of nasal sinus and cerebrum, and fixed them in 10% neutral-buffered formalin, processed routinely, and stained 5-μm sections with H&E. We performed a direct fluorescent antibody test at the OADDL rabies laboratory, following the protocol for postmortem rabies diagnosis, established by the Centers for Disease Control and Prevention (http://www.cdc.gov).^9^ To detect *T. gondii* DNA, we used a nested PCR assay that amplifies a portion of the *B1* gene, using 80 ng of DNA extracted from brain tissue.^8,24,25^ We included the RH strain of *T. gondii* isolated from cell culture as a positive control.^22^ The PCR products were resolved in agarose as described above.

The morphology of the specimens and the location within the host corresponded with the description for *S. chitwoodorum* in the literature.^12^ By PCR, we amplified a 629-bp fragment of the *COI* gene available in GenBank (PP195861). The sequencing results were 100% similar to a previous *S. chitwoodorum COI* sequence (MT454081.1).

The rostral and caudal olfactory bulbs had mild, acute, diffuse lymphohistiocytic meningitis with minimal neuropil perivascular cuffing (Suppl. Fig. 3). Gliosis and glial nodules in the frontal lobe, thalamus, and hippocampus were the same as those in the olfactory bulbs. Histologically, we observed mild chronic multifocal histiocytic and plasmacytic sinusitis with mucosal hyperplasia rostral to the cribriform plate, with multifocal eosinophilic, histiocytic, and lymphoplasmacytic sinusitis in the frontal sinus. On the cribriform plate and frontal sinus, we noted intraluminal nematodes (Fig. 2D). The adult nematodes were up to 1 mm in diameter, with a 25-µm thick, eosinophilic, smooth-to-undulating cuticle; celomyarian-polymyarian musculature; and pseudocelom containing intestinal and reproductive tracts. The female reproductive tracts contained larvae developing within eggs. Additionally, we observed various degrees of neuronal satellitosis and mononuclear perivascular cuffing, and Negri bodies in scattered neurons (Suppl. Figs. 4, 5).

Testing for RABV was performed upon submittal, and further testing was conducted following standard laboratory safety procedures. The direct fluorescent antibody test detected this skunk as positive for RABV; the *T. gondii* PCR assay did not detect DNA in the brain tissue. Skunks are the third most common host of RABV in the United States, after bats and raccoons,^19^ and are commonly submitted to diagnostic laboratories to be tested for rabies. In Texas and Oklahoma, skunks are considered the primary reservoir species for rabies.^19^ The skunk in our case was euthanized because of unusual behavior, tested for RABV, and identified as rabies-positive. The nematodes recovered from the cranium of the skunk in our case were observed incidentally and recovered during tissue collection. Parasites recovered were morphologically and molecularly identified as *S. chitwoodorum*, a member of the family *Metastrongylidae*.

Distribution of *S. chitwoodorum* has been linked to environments in which precipitation allows for populations of snail intermediate hosts to thrive and potentially increases survival of infectious first-stage larvae in the environment.^3^ Evidence of this parasite has been reported in the skulls of spotted and striped skunks from collections across the USA from 1895–1981, with damage ranging from none to severe, featuring holes of >7 mm.^11^ Molecular analysis of the *COI* gene of this parasite, mainly in striped skunks from Texas, have demonstrated that *S. chitwoodorum* has low genetic divergence.^3,13^

Histologic findings of meningitis in the olfactory bulbs and frontal lobe of the brain in our case are consistent with reports attributing similar lesions to the migration of *Skrjabingylus* spp.; neurologic deficits were reported to appear just 13 d post-infection.^15^ The skunk in our case had erratic behavior that led to the submission for RABV testing, although several etiologies may lead to neurologic signs in skunks. Behavioral alterations caused in skunks by *S. chitwoodorum* have not been detailed, but a 2018 report suggested that behavioral changes might be responsible for frequent submission by the public to test these skunks for RABV, with a 48.7% prevalence of *S. chitwoodorum* found in rabies-negative skunks.^13^ Because our case was positive for RABV, it is not possible to determine if the behavioral changes in this animal were caused by the viral pathogen alone or exacerbated by the presence of *S. chitwoodorum*. Behavioral changes that may be precipitated by the well-documented extensive skull damage by *S. chitwoodorum* to adult skunks may increase the risk of predation, accidental death, and lead to decreased ability to reproduce successfully; factors that may negatively impact overall skunk populations.^11^

In addition to RABV, *T. gondii* is another zoonotic pathogen for which we tested but did not detect. Skunks are often infected with *T. gondii* by ingestion of oocysts from the environment or by ingestion of infected small mammals, which may introduce an overlapping risk to skunks by exposure to *S. chitwoodorum* and *T. gondii*.^5,23^ This protozoan parasite has been described to alter behavior in its hosts, but information about *T. gondii* and changes in behavior in skunks is scarce.^4,6,10,21^ In experimental infections of skunks, *T. gondii* results in fatalities from acute disease 7–19 d post-infection.^21^ When *T. gondii* has been reported in skunks, bradyzoites have been found in multiple tissues including the brain, heart, liver, tongue, and skeletal muscles.^21^ However, these stages have been found more commonly within the myocardium than the brain in infected skunks.^21^ In our case, testing of myocardial tissue for *T. gondii* was not possible because only the cranium of the animal was submitted for rabies testing rather than the whole body.

Canine distemper virus (*Paramyxoviridae*, *Morbillivirus canis*) is another common differential cause of neurologic disease in skunks, and often causes clinical signs similar to those of rabies. Although distemper may pose a risk to canids and other wildlife, this virus is not considered zoonotic. Due to lack of zoonotic concern and additional tissue for testing, distemper virus testing was not pursued in our case. Although *S. chitwoodorum* is not zoonotic, other pathogens of skunks that may potentiate behavioral alterations, such as *T. gondii* and RABV, are transmissible to humans from wildlife species, with rabies constituting a significant fatality risk.

## Supplemental Material

sj-pdf-1-vdi-10.1177_10406387241293421 – Supplemental material for Skrjabingylus chitwoodorum in a rabies-positive striped skunk in Texas Supplemental material, sj-pdf-1-vdi-10.1177_10406387241293421 for Skrjabingylus chitwoodorum in a rabies-positive striped skunk in Texas by Sarah Myers, Brianne Taylor, Ragan Wilson, Shannon Caseltine and Ruth C. Scimeca in Journal of Veterinary Diagnostic Investigation
