# Supplementary material for: Skrjabingylus chitwoodorum in a rabies-positive striped skunk in Texas
Source: J Vet Diagn Invest. 2024 Nov 7;37(1):184–8. doi: 10.1177/10406387241293421 (PMC11559893; doi:10.1177/10406387241293421)
Supplement: sj-pdf-1-vdi-10.1177_10406387241293421 – Supplemental material for Skrjabingylus chitwoodorum in a rabies-positive striped skunk in Texas [file sj-pdf-1-vdi-10.1177_10406387241293421.pdf]

Myers S, et al. *Skrjabingylus chitwoodorum* in a rabies-positive striped skunk in Texas

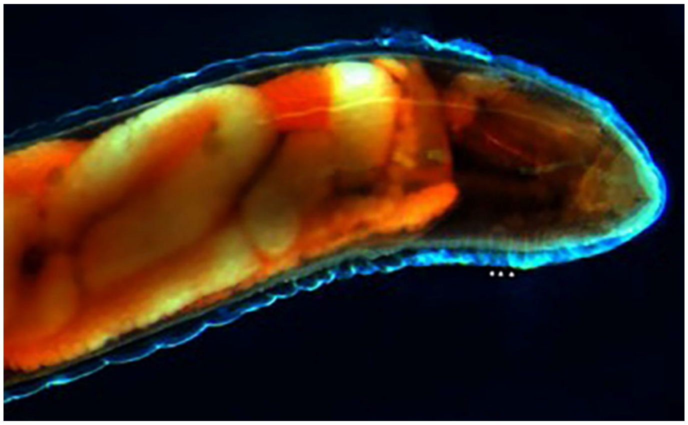

Supplemental Figure 1. *Skrjabingylus chitwoodorum* transparent cuticle with annular ridges (arrowheads).

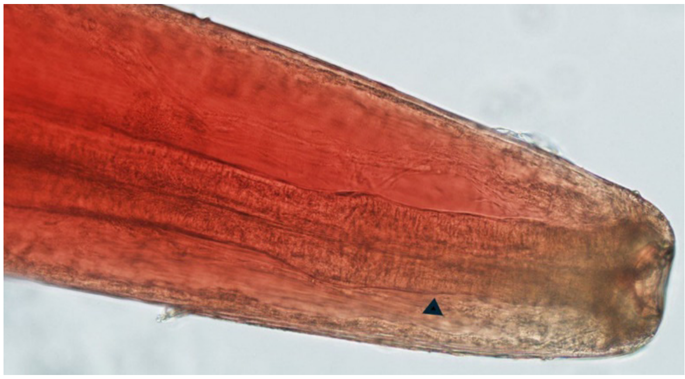

Supplemental Figure 2. *Skrjabingylus chitwoodorum* muscular esophagus (arrowhead).

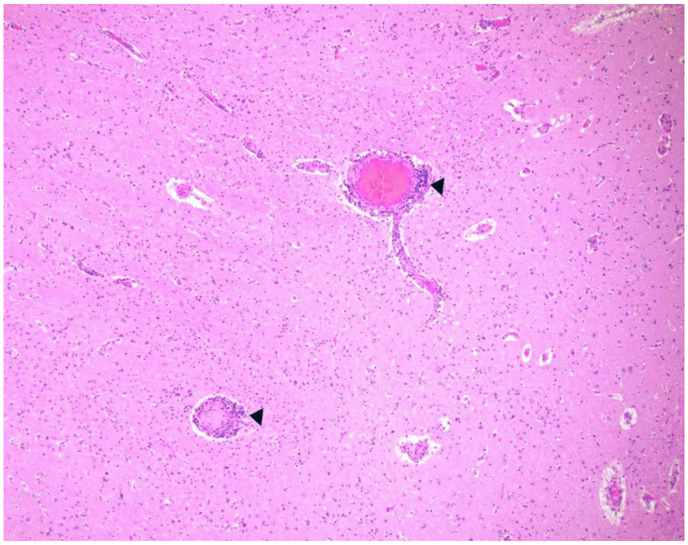

Supplemental Figure 3. Lymphohistiocytic perivascular cuffing (arrowheads).

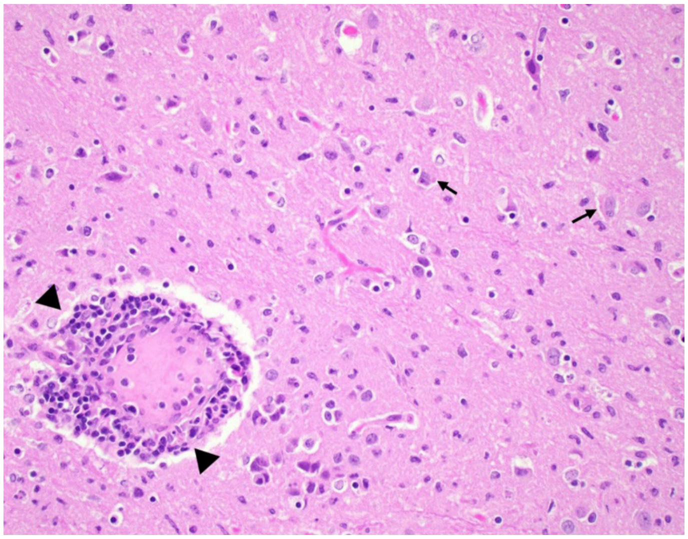

Supplemental Figure 4. Lymphohistiocytic perivascular cuffing (arrowheads). Neurons contain intracytoplasmic eosinophilic inclusion bodies (arrows).

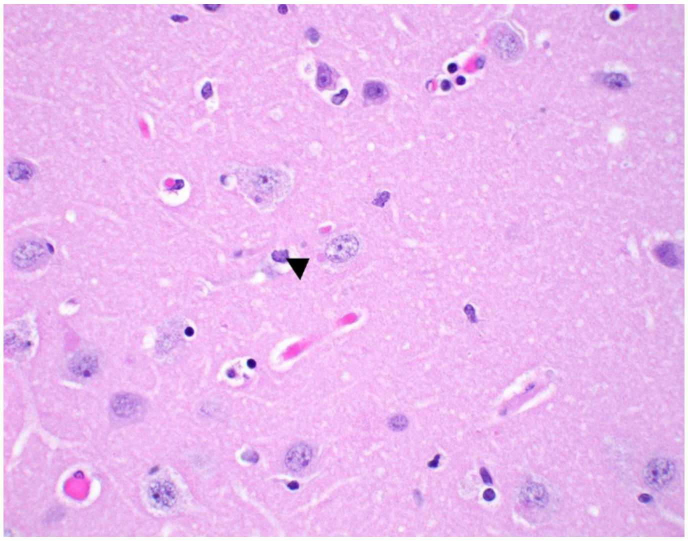

Supplemental Figure 5. Intracytoplasmic eosinophilic viral inclusion bodies (arrowhead) within a neuron.
